# In vitro antibacterial activity of poly (amidoamine)-G7 dendrimer

**DOI:** 10.1186/s12879-017-2513-7

**Published:** 2017-06-05

**Authors:** Mitra Gholami, Rashin Mohammadi, Mohsen Arzanlou, Fakhraddin Akbari Dourbash, Ebrahim Kouhsari, Gharib Majidi, Seyed Mohsen Mohseni, Shahram Nazari

**Affiliations:** 1 Department of Environmental Health Engineering, School of public Health, Iran University of Medical Sciences, Tehran, Iran; 2 Department of Life Science Engineering, Faculty of New Science and Technology, University of Tehran, Tehran, Iran; 3 Department of Microbiology, School of Medicine, Ardabil University of Medical Sciences, Ardabil, Iran; 4 Department of Materials science and Engineering, Tarbiat Modares University, Tehran, Iran; 5 Department of Microbiology, School of Medical, Iran University of Medical Sciences, Tehran, Iran; 6 Department of Environmental Health Engineering, School of public Health, Qom University of Medical Sciences, Qom, Iran; 7 Department of Environmental Health Engineering, School of public Health, Shahid Beheshti University of Medical Sciences, Tehran, Iran; 8 Department of Environmental Health Engineering, Developmental Center for Student Research and Technology Talent, School of Public Health, Iran University of Medical Sciences, Tehran, Iran

**Keywords:** Polyamidoamine-G7, Antibacterial activity, Gram-positive bacteria, Gram-negative bacteria, Cytotoxicity

## Abstract

**Background:**

Nano-scale dendrimers are synthetic macromolecules that frequently used in medical and health field. Traditional anibiotics are induce bacterial resistence so there is an urgent need for novel antibacterial drug invention. In the present study seventh generation poly (amidoamine) (PAMAM-G7) dendrimer was synthesized and its antibacterial activities were evaluated against representative Gram- negative and Gram-positive bacteria.

**Methods:**

PAMAM-G7 was synthesized with divergent growth method. The structural and surface of PAMAM-G7 were investigated by transmission electron microscopy, scanning electron microscope and fourier transform infrared. *Pseudomonas. aeruginosa* (*n* = 15), *E. coli* (*n* = 15), *Acinetobacter baumanni* (*n* = 15), *Shigella dysenteriae* (*n* = 15), *Klebsiella pneumoniae* (*n* = 10), *Proteus mirabilis* (*n* = 15), *Staphylococcus aureus* (*n* = 15) and *Bacillus subtilis* (*n* = 10) have been used for antibacterial activity assay. Additionally, representative standard strains for each bacterium were included. Minimum Inhibitory Concentration (MIC) was determined using microdilution method. Subsequently, Minimum Bactericidal Concentration (MBC) was determined by sub-culturing each of the no growth wells onto Mueller Hinton agar medium. The cytotoxicity of PAMAM-G7 dendrimer were evaluated in HCT116 and NIH 3 T3 cells by MTT assay.

**Results:**

The average size of each particle was approximately 20 nm. PAMAM-G7 was potentially to inhibit both Gram positive and gram negative growth. The MIC50 and MIC90 values were determined to be 2–4 μg/ml and 4–8 μg/ml, respectively. The MBC50 and MBC90 values were found to be 64–256 μg/ml and 128–256 μg/ml, respectively. The cytotoxity effect of dendrimer on HCT116 and NIH 3 T3 cells is dependent upon exposure time to and concentration of dendrimers. The most reduction (44.63 and 43%) in cell viability for HCT116 and NIH 3 T3 cells was observed at the highest concentration, 0.85 μM after 72 h treatmentm, respectively.

**Conclusions:**

This study we conclude that PAMAM-G7 dendrimer could be a potential candidate as a novel antibacterial agent.

## Background

Healthcare associated infection (HCAI) presents a major problem for patient safety and could lead to prolonged hospitalization, long-term disability, high costs for patients and their families, and excess deaths [1, 2]. In spite of the fact that modern medical science faces rapid advances, HCAIs still represent a worldwide public health issue and cause a significant additional financial burden for the health system. The amount of annual financial lost due to HCAIs for Europe and USA are 7 billion euro and 6.5 billion dollars, respectively [1, 3]. Multidrug-resistant (MDR) bacteria are reported as the main responsibility for treatment failure [4]. Methicillin resistant *Staphylococcus aureus* [5], extended-spectrum beta-lactamase-producing *E. coli* [6], carbapenem resistant *Acinetobacter buamanii* [4], and *Pseudomonas aeruginosa* [7] are the most important MDR’s which are associated with these infection.

**Fig. 1:**
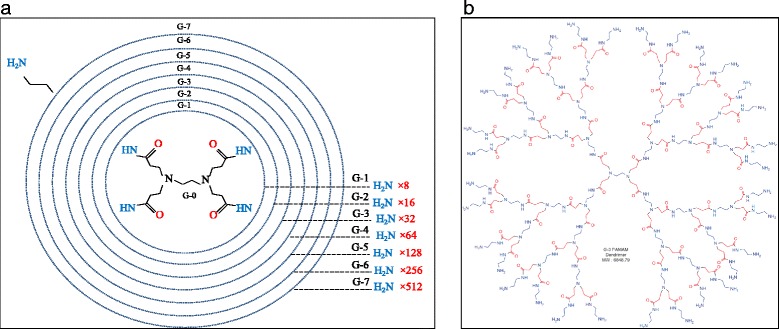
**a** conceptional scheme of PAMAM-G7 dendrimer; **b** structure of PAMAM-G3 dendrimer

Among *Shigella* species, *S. dysenteriae* is of particular importance, because: (1) *S. dysenteriae* produces the Shiga toxin and causes severe infections; (2) *S. dysenteriae* is associated with large dysentery epidemics in developing countries; and (3) *S. dysenteriae* strains isolated worldwide are often MDR, with plasmid-mediated resistance to commonly used antimicrobials such as trimethoprim-sulfamethoxazole, ampicillin, tetracycline and chloramphenicol [8, 9]. Therefore, increment in the number of nosocomial infection by strains of MDR, the discovery and development of novel antibacterial agents, particularly those with structures and mechanisms of action different from traditional antibiotics, and a low potential to induce antibiotic resistance, are needed more than ever in the control and treatment of HCAIs. Compared to bulk materials, Nanoparticles (NPs) may be strategically advantageous as active antimicrobial agents, since NPs are excellent adsorbents, catalysts, and sensors due to their large surface area and high reactivity [10]. In addition, current antibiotics generally targets three organs consists of: cell wall, translation machinery and DNA replication system [11]. Unfortunately, each one of these modes of actions is susceptible to bacterial resistance. Various simulation processes such as production of reactive oxygen species, electrostatic interaction with the cell membrane, ion release, internalization and etc. contribute to NPs mode of action [12]. Cationic nano dendrimers have emerged as promising novel antibiotic agents in recent years [13, 14]. Dendrimers are monodispersed, well-defined highly branched macromolecule, which exhibit an exponential increase in functional groups of each generation. Dendrimers have a highly branched three-dimensional architecture with spaces between the branches and since these empty spaces can accept guest molecules, various size of particle can be encapsulated by dendrimers [14, 15]. Due to the aforementioned properties, dendrimers have attracted great interest in exploring their potential biomedical applications such as drug delivery, gene transfection, and imaging [16, 17]. Recent research activities in this area include the study of antimicrobial activities of dendrimer derivatives. In most cases, biologically active agents can be encapsulated in the interior or, more often, tethered on the periphery of the dendrimers, therefore these dendrimers serve as carriers of biologically active agents. PAMAM dendrimers are the most extensively studied dendrimers. PAMAM dendrimers with a wide variety of functional groups at the periphery have the most antibacterial activity [15, 18]. Their high potency antibacterial activity attributed to the electrostatic interaction between the cationic dendrimer and the anionic bacteria cell surface so it can be caused cell death due to the disruption of lipid bilayer. Thus, dendrimer biocides may be very beneficial in terms of activity, localization in specific organs, reduced toxicity, and increased duration of action [16, 17]. The increasmen of generation of PAMAM dendrimers is followed by a double increase in the number of functional amine groups in the structure of dendrimer [15, 19]. We designed a conceptional scheme of PAMAM-G7 dendrimer which shows the number of functional amino groups in each generation (Fig. 1a). The structure of PAMAM-G3 is also displayed in Fig. 1b.

Regarding the fact that bacteria cause hospital infections, also considering the MDR bacteria, evaluation of antibacterial properties of PAMAM dendrimers and taking the advantage of their ability as an antibacterial and antiseptic can be a research priority. Present study aimed to determine antibacterial properties of PAMAM-G7 dendrimer using disc diffusion, broth microdilution (MIC and MBC determination) methods. To our knowledge, this is the first report on the inherent high antibacterial PAMAM-G7 dendrimer which is not modified with common antibacterial agents. Overall, all of the dominant bacteria which are the main cause of HCAIs are investigated in current study.

## Methods

### Bacterial strains

In this study 8 bacterial species including *Pseudomonas aeruginosa* (*n* = 15)*, E. coli* (*n* = 15), *Acinetobacter baumanni* (*n* = 15)*, Shigella dysenteriae* (*n* = 15)*, Klebsiella pneumoniae* (*n* = 10)*, Proteus mirabilis* (*n* = 15)*, Staphylococcus aureus* (*n* = 15) *and Bacillus subtilis* (*n* = 10) were selected. These bacteria have been isolated from clinical specimens and identified by conventional microbiological tests. Additionally, *E. coli* ATCC 25922, *P. aeruginosa* ATCC 27853, *A. baumannii* ATCC 17957, *S. dysenteriae* ATCC 13313, *K. pneumoniae* ATCC 1705, *P. mirabilis* ATCC 29906, *S. aureus* ATCC 25923 and *B. subtilis* ATCC 23857 were used as selected standard strains.

### Synthesis and characterization of poly (amidoamine)-G7 Dendrimer

Ethylenediamine (10 ml) was dissolved in 40 ml ethanol in a 1-l round-bottomed flask. Methyl acrylate (112 ml) was added at 40 °C and stirred for 30 h in the presence of nitrogen exposure. The Excessive methyl acrylate was removed under vacuum condition room temperature. A Michael addition between the amine and the acrylate yielded a product bearing four terminal methyl ester groups, defined as the G0.5 PAMAM. Subsequently, ethylenediamine (1.04 gmol) was dissolved in ethanol, was added to the G0.5 PAMAM. Then, a product bearing four terminal amino groups was obtained and defined as the G1 PAMAM, after stirring for 48 h in the presence of nitrogen and removing excess reactants by vacuum distillation, seventh generation PAMAM dendrimers was synthesized by repeating the above cycle [20].

The chemical formula of PAMAM-G7 is C_5102_H_10208_N_2042_O_1020_, molecular weight equal to 116,493 g/mol and the number of terminal amine groups is 512.

The molecular structure has clarify by Fourier transform infrared (FTIR, TENSOR 27 FTIR spectrometer, Bruker, Germany). Briefly, The samples were mixed with potassium bromide (KBr) powder and then the mixtures were made into pellet under high pressure. The sample pellet was scanned from 400 to 4000 cm^−1^. Pure KBr acted as blank. The Morphology and size distribution of PAMAM-G7 were analyzed by using transmission electron microscopy (TEM, Philips CM 30) and scanning electron microscope (SEM, Philips, XL30). For the TEM investigations, the samples were dispersed in ethanol and deposited by placing two drops of nanoparticle suspension onto carbon-covered copper-grids, followed by drying at room temperature. The structure of dendrimer was analyzed by scanning electron microscopy (SEM, Philips, XL30). The samples were prepared by mounting them on double sided carbon tape and coated with a thin layer of gold before imaging by sputtering method.

### Antibacterial activity assay

The antibacterial activity of PAMAM-G7 dendrimer was determined by using disc diffusion method against clinical and standard bacterial strains as listed above. The MIC and MBC of the agents were determined by micro dilution method according to CLSI procedure [21].

### Disc diffusion method

An overnight bacterial culture with the turbidity comparable to 0.5 Mc Farland turbidity standard was prepared. The surface of a Mueller Hinton agar plate (4 cm diameter) was cultured uniformly with a sterilized cotton swab. Dried filter paper discs (6 mm in diameter) containing 0.025, 0.25, 2.5 and 25 μg/disc were placed on the plate. Then, plates were incubated 24 h at 37 °C. The antibacterial activity was determined as the diameter of growth inhibition zone. In each plate a blank disc without the PAMAM-G7 was used as quality control.

### MIC and MBC testing

MIC testing was carried out by using a microdilution method based on the method recommended by the Clinical Laboratory Standards Institute [21]. 2-fold serial dilutions of PAMAM-G7 dendrimer were prepared in sterile Mueller Hinton Broth (MHB) for a testing concentration range of 1–256 μg/ mL. Then 100 μL from each dilution was transferred into the well of a microtiter plate and inoculated with 5 μL of standardized (1.5 × 10^7^ CFU/ mL) bacterial suspension. The microtiter plates were then incubated aerobically at 37 °C for 20–22 h, and the lowest concentration of the agent that inhibited visible growth was recorded as the MIC. One well was used as a positive control for the all tested samples (only media, inoculum, no PAMAM-G7). Furthermore, another well was used as a negative control (only media, no inoculum, no PAMAM-G7).

MBC was determined by sub-culturing 10 μL of broth from wells with no visible growth on nutrient agar plates. The number of colonies on agar were counted on a light board and compared to negative controls (only media, inoculum, no PAMAM-G7). The lowest concentration of PAMAM-G7 that killed 99.9% of the original inoculum was considered as MBC.

### Cytotoxicity assay

To analyze the cytotoxicity effect, we used MTT assay on HCT116 and NIH 3 T3 cells [22] (human intestinal cancer cell line) which were purchased from the Pasteur Institute Cell Bank of Iran (http://ncbi.pasteur.ac.ir/). Briefly, 5000 cells suspended in 96-well plates diluted in 100 μL RPMI 1640 media (Invitrogen, Carlsbad, CA) which were supplemented with 10% heat-inactivated fetal bovine serum (FBS, Invitrogen, Carlsbad, CA), and 100 mg/mL penicillin-streptomycin (Invitrogen, Carlsbad, CA) at 37 °C in a humidified atmosphere containing 5% CO_2_. After 24 h that all cells attached to the baseline, 100 μL of medium containing different concentration of PAMAM-G7 (0.043, 0.086, 0.17, 0.34, 0.515, 0.686 and 0.85 μM) were added to each determined well and incubated in above conditions for 48 h and 72 h. After these incubation, 20 μL containing 5 mg/ml MTT were added to the wells and incubated again for 4 h. During this time, the MTT (yellow tetrazolium salt) was enzymatically converted into the purple formazan precipitate by viable cells that the concentration of formazan shows the proportional of viable cells. Subsequently, all medium was aspirated from the cells, then added 150 μl DMSO to dissolve the formazan participates. Finally, absorbance was detected at 490 nm wavelength by ELISA plate reader. Controls were incubated for 48 and 72 h as a negative control and 0.002% benzalkonium chloride incubated for 30 min as a positive control.

### Statistical analysis

Statistical analysis of data was performed using the Mann-Whitney U test analysis and Analysis of Variance (ANOVA). Statistical significance was assumed at *P* < 0.05. Every experiment was repeated at least three independent times.

## Results

**Fig. 2:**
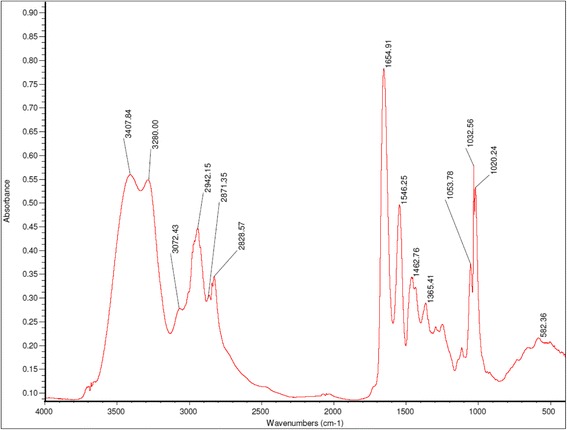
FTIR spectra of PAMAM-G7

The FTIR spectra of PAMAM-G7 dendrimer NPs were shown in Fig. 2. FTIR analysis of PAMAM-G7 NPs confirmed the existence of characteristic amides, terminal amino and etc. The data in Table 1 show the corresponding functional groups of the wavelengths indicated in Fig. 2.

**Table 1:** Band position of PAMAM-G7 spectrum

Band position(cm^−1^)	Assignment
1032.56	C–O stretching vibration
1654.91	C = O stretching (amide I)
1546.25	N-H bending/C-N stretching (amide II)
1462.78	H-C-H scissor
1365.41	H-C-H asymmetric
2828.57and 2942.15	C-H stretching
3407.84 cm^−1^and 3280	N-H stretching mode of amine I and amide groups

**Fig. 3:**
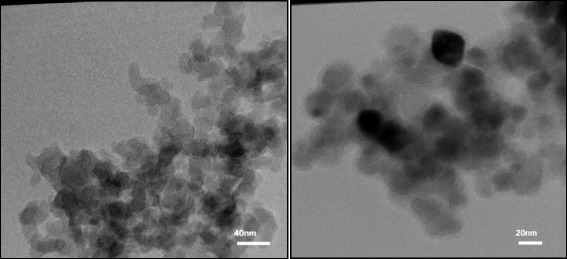
TEM Image of the PAMAM-G7 at different magnifications

**Fig. 4:**
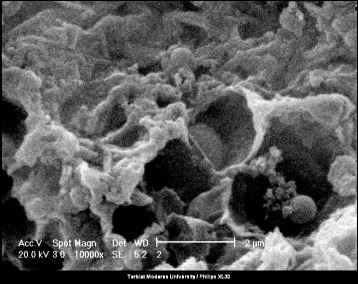
SEM Image of the PAMAM-G7

According to the FTIR spectra, the double peaks of NH_2_ groups are located at 3200–3500 cm^−1^ (NH stretching) and additional peaks at 1000–1350 and 1500–1630 cm^−1^ correspond to C–N stretching and NH bending, respectively. The amide A band (3407 cm^−1^) originates from a combination of amide II stretching and NH in-plane bending vibrations of the PAMAM-G7. Furthermore, the protonated carboxylic acids is characterized by absorption bands corresponding to a carbonyl stretch (C = O) between 1690 and 1750 cm^−1^, and C–OH vibrations between 1200 and 1300 cm^−1^. Upon deprotonation, the vibrational mode of C = O becomes coupled to that of the other deprotonated oxygen and its energy shifts to a lower energy level. This gives rise to an asymmetric stretching feature between 1550 and 1660 cm^−1^. The C–OH band also shifts to higher energies upon deprotonation, giving rise to a symmetric COO^−^ mode between 1300 and 1420 cm^−1^ as can be seen from the FTIR spectra. The TEM images of the PAMAM-G7 dendrimer NPs is shown in Fig. 3 PAMAM-G7 NPs were shown to have a spherical shape with a mean diameter size of 20 nm. Figure 4 shows SEM image of the PAMAM-G7 NPs. The SEM image demonstrates that PAMAM-G7 NPs are nearly spherical. It also displays multi-layered -like structure, which has smaller spherical substructures. The results of different concentrations of PAMAM-G7 dendrimer effect on isolated bacteria and standard strains (using disc diffusion method) are shown in Tables 2 and 3, respectively. According to the obtained results, PAMAM-G7 dendrimer actively inhibited the growth of isolated Gram-negative and Gram-positive bacteria and standard strains. However, the antibacterial activity of PAMAM-G7 on the isolated bacteria was less than that on the standard strains.

**Table 2:** The mean and confidence interval for inhibition zone diameter of isolated bacteria VS different PAMAM-G7 dendrimer concentrations

Dendrimer concentration, μg/disc	Zone of inhibition, mm, [Mean (CI)]							
	*E. coli*	*A.baumannii*	*K. pneumoniae*	*S. dysenteriae*	*P. aeruginosa*	*P. mirabilis*	*B. subtilis*	*S. aureus*
0.025	0	0	0	0	0	10 (8.16, 11.84)	0	10 (8.16, 11.84)
0.25	0	0	17 (16.08, 17.92)	0	0	19 (16.23, 21.44)	9 (8.08, 9.92)	11 (8.56, 13.44)
2.5	0	0	19 (18.08, 19.92)	14 (12.16, 15.84)	0	22 (19.56, 24.44)	9 (7.4, 10.6)	11 (7.8, 14.2)
25	22 (20.4, 23.6)	13 (11.6, 14.4)	27 (24.56, 29.44)	30 (27.56, 32.44)	24 (21.23, 26.77)	33 (31.4, 34.6)	18 (16.4, 19.6)	22 (19.56, 24.44)

**Table 3:** The mean and confidence interval for inhibition zone diameter of standard strains VS different PAMAM-G7 dendrimer concentrations

Dendrimer concentration, μg/disc	Zone of inhibition, mm, [Mean (CI)]							
	*E. coli* ATCC 25922	*A. baumannii*ATCC 7,957	*K. pneumoniae* ATCC 49131	*S. dysenteriae* ATCC 13313	*P. aeruginosa* ATCC27853	*P. mirabilis* ATCC 29906	*B. subtilis* ATCC 23857	S. aureus ATCC 25923
0.025	0	0	0	0	0	12 (10.6, 12.7)	0	12 (10.6, 13.41)
0.25	0	10 (9, 10.9)	20 (16.77, 23.33)	15 (13.4, 16.6)	0	21 (18.7, 22.58)	9 (7.59, 10.41)	13 (12, 13.9)
2.5	10 (9, 10.9)	14 (13, 14.9)	21 (20.08, 21.92)	20 (18.6, 20.72)	9 (8.08, 9.92)	24 (22.2, 25)	10 (8.9, 11)	19 (17.6, 20.4)
25	26 (25, 26.9)	20 (17.2, 22.8)	29 (28.08, 29.92)	32 (30.6, 33.4)	25 (22.7, 26.5)	36 (35.08, 36.9)	22 (21, 22.9)	31 (30.47, 31.53)

It was also a statistically significant difference (*p‹0.05*). The most of sensitivity related to *P. mirabilis* ATCC 29906*, S.* dysenteriae ATCC 13313 *and S. aureus* ATCC 25923 at concentration of 25 (μg/disc) PAMAM-G7 with zone of inhibition 36, 32 and 31 mm, respectively. In addition, the least sensitivity is related to *A. baumannii* at 25 (μg/disc) concentration of dendrimer with 13 mm zone of inhibition. The concentration of 0.025 (μg/disc) dendrimer had no effect on the studied bacteria, except *P. mirabilis and S. aureus*.

The MIC50 and MIC90 values for all selected bacteria were determined 2–4 μg/ml and 4–8 μg/ml respectively (Table 4). The MBC50 and MBC90 values were found to be 64–256 μg/ml and 128–256 μg/ml, respectively (Table 5). The highest MIC50 and MIC90 values for the clinical isolates were 4 and 8 μg/ml, respectively (Table 4). This was found in *E. coli*, *A. baumannii*, *P. aeruginosa* and *S. aureus* isolates.

**Table 4:** MIC of the PAMAM-G7 dendrimer for isolated bacteria

Bacteria spp	MIC (μg/ml) n(%)				MIC 50	MIC 90
	1	2	4	8		
*E. coli*			10 (66.7)	5 (33.3)	4	8
*A. baumannii*			9 (60)	6 (40)	4	8
*K. pneumoniae*			7 (70)	3 (30)	2	4
*S. dysenteriae*	5 (33.3)	10 (66.7)			2	2
*P. aeruginosa*			12 (80)	3 (20)	4	8
*P. mirabilis*	3 (20)	12 (80)			2	2
*B. subtilis*		1 (10%)	9 (90)		4	4
*S. aureus*			10 (66.6)	5 (33.3)	4	8

**Table 5:** MBC of the PAMAM-G7 dendrimer for isolated bacteria

Bacteria spp	MBC (μg/ml) n(%)			MBC 50	MBC 90
	64	128	256		
*E. coli*		3 (20)	12 (80)	256	256
*A. baumannii*		9 (60)	6 (40)	128	256
*K. pneumoniae*		7 (70)	3 (30)	128	256
*S. dysenteriae*	9 (60)	6 (40)		64	128
*P. aeruginosa*		2 (13)	13 (87)	256	256
*P. mirabilis*	3 (20)	12 (80)		128	128
*B. subtilis*	1 (10)	9 (90)		128	128
*S. aureus*		5 (33)	10 (67)	256	256

Table 4 also shows that the least amount of MIC50 and MIC90 were related to *S. dysenteriae* and *P. mirabilis* (2 μg/ml). Table 5 shows that the highest MBC50 and MBC90 values were related to *E. coli*, *P. aeruginosa* and *S. aureus* (256 μg/ml). Moreover, it can be seen from Table 5 that the least amount of MBC50 and MBC90 with 64 and 128 μg/ml, respectively, are of the bacteria *S. dysenteriae.* In addition, the MIC and MBC of the standard bacteria were also studied. MIC was found to be 4 μg/ml for *E. coli* ATCC 25922, *A. baumannii* ATCC 17957, *P. aeruginosa* ATCC27853, *S. aureus* ATCC 25923 and 2 μg/ml for *K. pneumoniae* ATCC 49131, *S. dysenteriae* ATCC 13313, *P. mirabilis* ATCC 29906 and *B. subtilis* ATCC 23857. Besides, MBC for *E. coli* ATCC 25922, *A. baumannii* ATCC 17957, *P. aeruginosa* ATCC27853, *S. aureus* ATCC 25923 and *K. pneumoniae* ATCC 49131 was 128 μg/ml and for *S. dysenteriae* ATCC 13313, *P. mirabilis* ATCC 29906 and *B. subtilis* ATCC 23857 was seen to be 64 μg/ml.

**Fig. 5:**
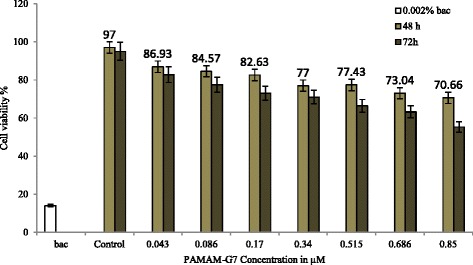
PAMAM-G7 cytotoxicity to HCT116 cells measured by MTT survival assay in 48 h and 72 h with 0.002% benzalkonium chloride (bac) as the positive control. Percent survival of HCT116 cells upon treatment with PAMAM-G7 at various concentrations is based on an untreated control. The data show the mean from two separate experiments (48 h and 72 h) with three replicates per condition, and the error bar represents a standard deviation

**Fig. 6:**
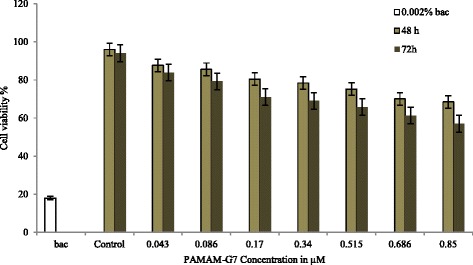
PAMAM-G7 cytotoxicity to NIH 3 T3 cells measured by MTT survival assay in 48 h and 72 h with 0.002% benzalkonium chloride (bac) as the positive control. Percent survival of NIH 3 T3 cells upon treatment with PAMAM-G7 at various concentrations is based on an untreated control. The data show the mean from two separate experiments (48 h and 72 h) with three replicates per condition, and the error bar represents a standard deviation

The profiles of cytotoxicity of PAMAM-G7 against HCT116 and NIH 3 T3 cells after 48 and 72 h treatment are shown in Figs. 5 and 6, respectively.

Figures 5 and 6 also indicate that, by the increase of concentration and exposure time to PAMAM-G7 dendrimer, cytotoxicity gradually increases. Moreover, statistical results of ANOVA showed that in the exposure times of 48 and 72 h, the cytotoxicity of PAMAM-G7 dendrimer in all the concentrations and towards both cell lines was significantly increased in comparison to the negative control (*P* < 0.05). So, by the increase of PAMAM-G7 dendrimer concentration from 0.043 to 0.85 μM, cell viability (HCT116) declined after 48 and 72 h from 86.93 to 70.66% and 82.8 to 55.37%, respectively. Also for NIH 3 T3 cells in the same conditions, cell viability declined after 48 and 72 h from 87.63 to 68.5% and 83.9 to 57%, respectively. However, there are relatively low cytotoxicity effects of PAMAM-G7 with different concentrations towards HCT 116 and NIH 3 T3 cells during 48 and 72 h, where no IC50 value was obtained. Also, in the range of MIC value the cytotoxicity of PAMAM-G7 on both cell lines was relatively low.

## Discussion

Today modern hospitalization is the occurrence of nosocomial or healthcare acquired infections caused by MDR pathogens [23]. PAMAM dendrimers have been investigated for their biological applications, but antibacterial activity has not been extensively explored [17, 24]. In this work, we used disc diffusion and broth microdilution (MIC and MBC determination) methods to assess the antibacterial activity of PAMAM-G7 against eight most common human pathogens isolated from clinical specimens, as well as standard strains. *S. aureus* is a microorganism causing a wide range of infections from local infections of skin and soft tissue to pneumonia and endocarditis [25]. *A. baumannii* is an important human pathogen causing hospital-acquired infections, such as ventilator associated pneumonia, bacteremia, meningitis, urinary tract, and wound infections [26]. *E. coli* strains are responsible for several forms of diarrheal disease [27] and meningitis in neonates [28]. *P. aeruginosa* is inherently resistant to drugs because of its less permeable cell wall and a variety of efflux pumps [29]. *K. pneumoniae* can be found commonly in humans and animals’ mouth, skin, and intestines as an opportunistic pathogen, frequently causing pneumonia, infection of the urinary tract, and soft tissues [30].

The infections caused by *S. dysenteriae* and *Proteus mirabilis* had a serious clinical problem. *S. dysenteriae* is the cause of brisk and deadly epidemics and pose major health problems in the poorest populations. Alternative therapeutic strategies are necessarily in search due to the emergence of MDR in Shigellae [31]. Antibacterial properties of PAMAM-G7 dendrimer was checked also against *B. subtilis*. *B. subtilus* is not a common HCAI organism, but it was used as a representative for spore-forming bacteria [32]. The size of the inhibition zone clearly shows that with increasing concentrations of the PAMAM-G7 NPs, the surrounding zone of the discs is expanded (Tables 2 and 3). The results of this study showed that very low concentrations (0.025 μg/disc) of the dendrimer PAMAM-G7 inhibits the growth of the *P. mirabilis* and *S. aureus* isolates (Tables 2 and 3). The study carried out by Izanloo et al. [33], by disc diffusion method on the effect of dendrimer PAMAM-G4 on *E. coli*, *Enterobacter cloacae*, *B. subtilis* and *S. aureus,* concluded that concentration of 0.05 μg/disc has no effect on these bacteria and it was shown that the antibacterial effect of PAMAM-G4 takes place in higher concentrations. Izanloo et al. [34], in another study which was carried out by disc diffusion method and was aimed to evaluate the effect of PAMAM-G4 dendrimer on *Klebsiella oxytoca*, *P. mirabilis* and *P. aeruginosa,* has showed that concentrations of 0.5, 5 and 50 μg/disc of PAMAM-G4 has no effect on these selected bacteria. Probably, the higher antibacterial effect of PAMAM-G7 dendrimer in comparison to lower generation dendrimers can be attributed to high density, ordered, hyper branching structure, high spatial void between branches, large number of terminal functional groups and relatively large molecular size of PAMAM-G7 [24]. These characteristics lead to high surface area in dendrimer PAMAM-G7 which causes higher activity of dendrimers in surface of culture and higher efficiency at lower concentrations. Figure 4 shows a SEM image of dendrimer PAMAM-G7. Because of their multi-layered structures with high purity, they can trap and absorb many microbial agents. Dendritic structures known as dendriform with progressive structure are illustrated in this figure. Too many branches of this dendriform lead to increase in the dendrimer surface area therefore they absorb microbes on their surface. On the other hand, nano holes created between branches trap biological agents and destroy them. But most importantly, it is the number of terminal amine groups, which for generation 4 is 64, while the number of terminal amine groups for PAMAM-G7 is 512 (http://www.dendritech.com/pamam.html) [35]. These functional groups are adsorbed on the bacterial cell surfaces, diffused through the cell wall, bonded to cytoplasmic membrane and release electrolytes such as potassium ions and phosphate from the cell, also nucleic materials such as DNA and RNA due to disruption and disintegrate of the cytoplasmic membrane. Therefore it is proposed that the antibacterial property of dendrimers is mediated by disrupting the bacterial outer and inner membrane by terminal amine groups [17, 36].

According to MIC or MBC values (Tables 4 and 5), it is clear that PAMAM-G7 has antibacterial effects and can be used as antibacterial agent. Previous studies have been shown that these antibacterial agents cause bacterial cell membrane damage, spatial deformation, degradation of bacterial enzymes, damage of chromosome and bacteria cell wall damage [15, 37]. This character refers to end amine groups in dendrimer structure which interact with negative charge of membrane or microorganism cytoplasm, causing bacterial cell wall damage and finally, inactivation of bacteria [16]. Figure 2 shows the FTIR spectra of PAMAM-G7 dendrimer. As shown, 9 peaks are detectable at 1032 cm^−1^, 1365 cm^−1^, 1462 cm^−1^, 1546 cm^−1^, 1654 cm^−1^, 2828 cm^−1^, 2942 cm^−1^ 3280 cm^−1^ and 3407 cm^−1^which the last peak is related to N-H stretching vibration of primary amine. Other main band positions, based on wavenumber, and their assignments are presented in Table 1.

Thus, the PAMAM-G7 dendrimer is an efficient antibacterial agent for both Gram-negative and Gram-positive bacteria. Chen et al. [38], observed the antibacterial effect of polypropyleneimine dendrimer modified with quaternary ammonium groups on Gram-positive and Gram-negative bacteria. Likewise, Xue. et al. and Charles. et al., have shown that amino-terminated PAMAM-G2 and G3 dendrimers possess significant antibacterial effects on MDR strains [14, 15].

As shown in Tables 2, 3, 4 and 5, the *E. coli, P. aeruginosa, A. baumannii and S. aureus* had a higher resistance than other studied bacteria. Probably the effect of lower concentrations of dendrimer PAMAM-G7 on the bacteria such as *E. coli, P. aeruginosa, A. Baumannii* and *S. aureus* than other target bacteria can be due to intrinsic resistance of these bacteria [5, 39, 40].

Also, the cytotoxicity of PAMAM-G7 was investigated on HCT 116 and NIH 3 T3 cell lines (mammalian cells). The obtained data (Figs. 5 and 6) show that by increasing both concentration and exposure time the cytotoxity effect on target cells increases.

Mukherjee et al. [22] indicated different generations (G4, G5 and G6) of PAMAM dendrimers with variety of doses which increasing dose and generation of these dendrimers cause decrease in the percentage of healthy and early apoptotic cell which increasing dose and generation of these dendrimers cause decrease in the percentage of healthy and early apoptotic cell. At high concentrations, PAMAM can lead to the formation of nanoscale holes in eukaryotic membranes [22, 41, 42]. Highly branched cationic polymers permeate eukaryotic membranes better than linear molecules, such as LL-37 [42], so PAMAM dendrimers are more toxic for eukaryotic cells. This property of branched polymers has been widely used in foreign gene or drug transfaction in eukaryotic cells [17]. The charge density on the polymer also plays an important role in permeability [42]. After 72 h of treatment at the highest concentration of PAMAM-G7 (0.85 μM), 55.37 and 57% of HCT116 and NIH 3 T3 cells survived, respectively (Figs. 5 and 6). The value of obtained MIC50, MIC90 (2–4 and 4–8 μg/ml, respectively) and MIC for standard strains (2 μg/ml) for both Gram-positive and Gram-negative bacteria, showed, PAMAM-G7 at relatively lower concentrations, has high toxic effect on both Gram-negative and Gram-positive bacteria.

However in 0.086 μM (10 μg/ml) PAMAM-G7 and after 48 and 72 h, 84 and 77% of HCT 116 cells were survived, respectively (Fig. 5). Also in same condition 85.61 and 79.24% of NIH 3 T3 cells were survived, respectively (Fig. 6).

Furthermore the most important attachment is great toxicity of PAMAM-G7 on Gram-negative and Gram positive bacteria. However PAMAM-G7 has relatively low toxicity on HCT 116 and NIH 3 T3 cells. To further elucidate this observation, it was noted that the polycationic PAMAM molecules prefer to bind to bacteria cells that carry a higher density of negative charges on their surfaces rather than eukaryote cell lines.

Initial electrostatic interaction, followed by further interactions, including hydrophobic interactions between the dendrimers and cell membrane, are shown to be necessary to cause cell lysis, since it has been shown that surfaces presenting a high density of amino groups have no marked effect on the membrane of the attached bacteria [17, 42].

However, various studies have shown that, PAMAM dendrimers show relatively high toxicity against various eukaryotic cells. But the notable point is that, most studies regarding the use of PAMAM dendrimers, are in the field of drug delivery and gene transfection. The high amount of dendrimers is required on drug and gene delivery so, such studies indicate the high toxic effect of PAMAM dendrimers on eukaryotic [43, 44], the cytotoxicity of PAMAM dendrimers, often investigates in higher concentrations. In a study the toxic effect of different PAMAM dendrimers (G3, 3.5, 4, 4.5, and) generations on HepG2 and DU145 cell lines was investigated. It indicate, HepG2 were less sensitive than DU145 cells with IC50 values ≥ 402 μM (PAMAMs) and ≤13.24 μM (PAMAMs) for DU145 [45]. In another study the cytotoxicity of various PAMAM dendrimers (G4, G5, G6) generations on HaCaT and SW480 cells was measured by MTT assay, after 24 h exposure it was found that, EC50 concentrations for PAMAM G4, G5 and G6 in SW480 cells were equal to 1.44, 0.37 and 1.16 μM, respectively, and for HaCaT cells were equal to 1.02, 1.07, and 3.21 μM, respectively [22].

## Conclusion

The results of this study showed PAMAM-G7 dendrimer has good antibacterial activity against both Gram-positive and Gram-negative bacteria. We think this compound can be implemented as an antiseptic and disinfectant agent in health sections. In addition, in the MIC value range, the cytotoxicity effect of PAMAM-G7 on HCT 116 and NIH 3 T3 cells was relatively lower. So, PAMAM-G7 could be an excellent candidate as new class of antibacterial compounds for control of bacterial infections. However to achieve this goal further studies are needed.

## Abbreviations

CLSI: Clinical and laboratory standards institute
FTIR: Fourier transform infrared
HCAI: Healthcare associated infection
IROST: Iranian research organization for science and technology
MBC: Minimum bactericidal concentrations
MDR: Multi-drug resistant
MIC: Minimum inhibitory concentration
NPs: Nanoparticles
PAMAM-G7: Polyamidoamine-G7
SEM: Scanning electron microscope
TEM: Transmission electron microscopy
